# Indigenous Peoples of North America: Environmental Exposures and Reproductive Justice

**DOI:** 10.1289/ehp.1205422

**Published:** 2012-08-16

**Authors:** Elizabeth Hoover, Katsi Cook, Ron Plain, Kathy Sanchez, Vi Waghiyi, Pamela Miller, Renee Dufault, Caitlin Sislin, David O. Carpenter

**Affiliations:** 1 Ethnic and American Studies, Brown University, Providence, Rhode Island, USA; 2 Running Strong for American Indian Youth, Alexandria, Virginia, USA; 3 Aamjiwnaang First Nation, Sarnia, Ontario, Canada; 4 Tewa Women United, Santa Cruz, New Mexico, USA; 5 Native Village of Savoonga, Alaska, USA; 6 Alaska Community Action on Toxics, Anchorage, Alaska, USA; 7 Food Ingredient and Health Research Institute, Naalehu, Hawaii, USA; 8 Women’s Earth Alliance, Berkeley, California, USA; 9 Institute for Health and the Environment, University at Albany, Rensselaer, New York, USA

**Keywords:** Alaska Natives, environmental justice, First Nations, Native Americans, reproductive justice

## Abstract

Background: Indigenous American communities face disproportionate health burdens and environmental health risks compared with the average North American population. These health impacts are issues of both environmental and reproductive justice.

Objectives: In this commentary, we review five indigenous communities in various stages of environmental health research and discuss the intersection of environmental health and reproductive justice issues in these communities as well as the limitations of legal recourse.

Discussion: The health disparities impacting life expectancy and reproductive capabilities in indigenous communities are due to a combination of social, economic, and environmental factors. The system of federal environmental and Indian law is insufficient to protect indigenous communities from environmental contamination. Many communities are interested in developing appropriate research partnerships in order to discern the full impact of environmental contamination and prevent further damage.

Conclusions: Continued research involving collaborative partnerships among scientific researchers, community members, and health care providers is needed to determine the impacts of this contamination and to develop approaches for remediation and policy interventions.

American Indian (AI) and Alaska Native (AN) peoples compose 1.7% of the population of the United States (Census Briefs 2012), and First Nations, Metis, and Inuit peoples compose 3.8% of the population of Canada (Statistics Canada 2006). Although these groups differ markedly in some aspects of culture and lifestyle, they unfortunately suffer from many common problems. Rates of poverty, unemployment, substance abuse, and violence are high, and overall life expectancy for indigenous people is less than that among whites. Mortality rates for AN populations are 60% higher than those of the U.S. white population (Day and Lanier 2003), and mortality rates in AI populations are about twice that of the general U.S. population (Kunitz 2008). AI/AN adults (16.1%) were more likely than black adults (12.6%), Hispanic adults (11.8%), Asian adults (8.4%), or white adults (7.1%) to have ever been told they had diabetes. These rates vary by region, from 5.5% among AN adults to 33.5% among AI adults in southern Arizona (Centers for Disease Control and Prevention 2011). Similarly, rates of diabetes among indigenous populations in Canada are 3–5 times higher than the general population (Sharp 2009). In addition, AI/AN have the lowest cancer survival rates among any racial group in the United States (U.S. Commission on Civil Rights 2004).

Many health conditions in indigenous communities are attributed to poverty, lifestyle, genetics, and an inadequate health care delivery system, but in many cases they are also compounded by exposure to environmental contaminants. These exposures affect not only current community residents, and those born into these exposed communities, but also generations to come. Exposure to environmental contaminants can increase these health risks for both the mother and her unborn children. Exposure of the unborn to environmental chemicals such as methylmercury, pesticides, and polychlorinated biphenyls (PCBs) not only increases the risk of developing several diseases later in life (Grandjean 2008) but also results in impairment of intellectual function for life (Carpenter 2006). In this commentary we will explore the linkages between environmental and reproductive health and justice issues in Native North American communities.

## Environmental and Reproductive Health and Justice

The U.S. Environmental Protection Agency (EPA 2012) defines environmental justice as

the fair treatment and meaningful involvement of all people regardless of race, color, national origin, or income with respect to the development, implementation, and enforcement of environmental laws, regulations, and policies.

In tribal communities in the United States, environmental mitigation is significantly behind that of nontribal communities (U.S. EPA 2004). The situation is equally concerning for indigenous communities in Canada, where legislation that deals directly with the inequalities created by environmental injustice is for the most part nonexistent (Dhillon and Young 2010). As Mascarenhas (2007) observed,

whether by conscious design or institutional neglect, Native American communities face some of the worst environmental devastation in the nation.

Sites ranging from industry to mining to military bases, as well as the release of pesticides and other agricultural by-products, negatively affect not only the surrounding environment, but the health, culture, and reproductive capabilities of the communities they border. Because of subsistence lifestyles, spiritual practices, and other cultural behaviors, tribes have multiple exposures from resource use that could result in disproportionate environmental impacts (U.S. EPA 2004).

Reproductive justice is

the right to have children, not have children, and parent the children we have in safe and healthy environments—[and] is based on the human right to make personal decisions about one’s life, and the obligation of government and society to ensure that the conditions are suitable for implementing one’s decisions. (SisterSong 2012)

As such, reproductive justice, a term that has not yet appeared in the environmental health literature, embeds reproductive rights in an intersectional framework that includes social justice and human rights (Luna 2010). Reproductive justice stresses both individual and group rights because the ability of a woman to determine her reproductive destiny is in many cases directly tied to conditions in her community (Shen 2006). The concept of environmental reproductive justice involves ensuring that a community’s reproductive capabilities are not inhibited by environmental contamination.

In the case studies we highlight below, struggles for environmental and reproductive justice have often converged as communities have become concerned about the impact of environmental contamination on their ability to reproduce and create culturally competent tribal citizens. These issues were explored in July 2011 in an Environmental Reproductive Health Symposium and Retreat organized by the First Environment Collaborative in Hot Springs, South Dakota, near the homeland of the Lakota Sioux.

The focus of this meeting was to explore the common issues of exposure to environmental contaminants and the health consequences of this exposure. The intent was to facilitate and nurture partnerships among the indigenous community organizations, researchers, scientists, and health care providers. The recommendations that came from the symposium include the need for additional community-based research that will support efforts to achieve environmental reproductive justice, and the need to support policy regulations that will better protect indigenous communities from both local and more widespread sources of environmental contamination. Below we present the environmental and reproductive health issues faced by each of the indigenous communities who were represented at this symposium, and discuss the need to develop the concept of environmental reproductive justice.

## Aamjiwnaang

Perhaps the most strikingly contaminated community is that of the Aamjiwnaang near Sarnia, Ontario, Canada, a 12-km^2^ reserve that is home to about 850 Anishnaabe First Nations people. The reserve is surrounded by 62 major industrial facilities located within 25 km, including oil refineries, chemical manufacturers (40% of Canada’s chemical industry), and manufacturers of plastics, polymers, and agricultural products. The area is known as “Chemical Valley.” Levels of air pollutants, including volatile organic compounds, are high (Atari and Luginaah 2009). In 1996, hospital admissions for women in Chemical Valley were 3.11 times the expected rates for women and 2.83 times those for men than would be expected based on other rates for Ontario. These admissions were especially pronounced for cardiovascular and respiratory ailments, and were hypothesized to be pollution related (Fung et al. 2007). About 40% of Aamjiwnaang residents require use of an inhaler, and 17% of adults and 22% of children are reported to have asthma (MacDonald and Rang 2007). The ratio of male births declined over the period 1984–1992 from > 0.5 to about 0.3, a change that may at least partly reflect effects of chemical exposures (Mackenzie et al. 2005.) Releases of chemicals have also interfered with the community’s cultural life, affecting hunting, fishing, medicine gathering, and ceremonial activities (MacDonald and Rang 2007).

## St. Lawrence Island (SLI)

The SLI Yupik live in two villages of about 800 people each. SLI, the largest island in the Bering Sea, lies just 240 km south of the Arctic Circle and is distant from industrial contamination sources. However, the Arctic acts as a “cold trap” and is a hemispheric sink for persistent organic pollutants (POPs) that are transported through a process known as global distillation via atmospheric transport from warmer regions (Wania 2003). In addition, there are two abandoned military sites on the island that contain fuels, pesticides, PCBs, metals, and solvents.

POPs bioaccumulate and biomagnify in the lipid-rich Arctic food webs, some to dangerous levels. The rendered oils of bowhead whale, seals, and walrus contain PCB concentrations of 193–421 ppb (Welfinger-Smith et al. 2011). For reference, the U.S. EPA risk-based consumption limit for PCBs in fish to avoid excess risk of cancer is 1.5 ppb (Welfinger-Smith et al. 2011). Rendered oils, blubber, and other fatty tissues from marine mammals are critical components of the traditional diet that provide important nutritional and cultural benefits. Blood serum of the Yupik people contains PCB levels 4–12 times higher than that of the general U.S. population. The predominant source is global transport; however, the former military site at Northeast Cape contributes to the PCB exposure (Carpenter et al. 2005). Although traditional foods are the primary source of exposure to POPs, harvest and consumption of these foods is a defining attribute of the SLI Yupik way of life—a necessary part of maintaining cultural identity. Although a systematic health study has not been done in this community, the residents believe they suffer from excess rates of cancer, thyroid disease, diabetes, and cardiovascular and other chronic diseases (Carpenter et al. 2005; Henifin 2007). This was most forcefully stated in a film interview of Annie Alowe, a SLI woman dying of breast cancer who believed it was caused by the chemical contamination (Miller and Riordan 1999).

## Tewa Pueblo

In addition to deposition of petrochemical and military waste, mining tends to heavily impact native communities. Uranium mining and mine tailings are major problems in both South Dakota and New Mexico. There was extensive uranium mining in the Southwest in the past, often on Indian land, and the mounds of mine tailings leached uranium into drinking and groundwater (Landa and Gray 1995). Uranium is both radioactive and has direct metal toxicity, which results in increased risk of cancer, birth defects, and kidney disease (Craft et al. 2004). In addition to mining effluents, the Tewa community in northern New Mexico is also exposed to toxic and radioactive wastes coming from releases from the Los Alamos National Laboratory, spread by air and surface and groundwater. Although a systematic health study has not been conducted in these populations, some environmental testing has been commissioned by local nonprofit organizations, which found PCB levels 25,000 times the standard for human health and 1,000 times over the standard for wildlife habitat in Los Alamos Canyon (Amigos Bravos and Concerned Citizens for Nuclear Safety 2006). Amigos Bravos won a settlement in May 2011 against the U.S. EPA and Los Alamos over discharge permits that will require clean up of a number of sites, increase monitoring, and install pollution control measures (van Buren 2011). However, these measures do little to determine the impact this contamination has had on the health and culture of the region’s residents.

## Oglala Lakota, Pine Ridge

Although starkly beautiful in landscape and home to myriad artists and storytellers, the Pine Ridge Indian Reservation in South Dakota, home to 25,000 Oglala Lakota people, is notoriously poverty stricken. Forty-nine percent of the residents live below the federal poverty level, and the infant mortality rate is five times higher than the national average (Ruffin 2011). Native Americans in the Northern Plains region have a cancer mortality rate approximately 40% higher than that of the overall population (Rogers and Petereit 2005). Although these health disparities are often attributed to the intense poverty in this region, since the late 1970s community organizations like Women of All Red Nations (WARN) have suspected links between Lakota health issues and the region’s history of uranium mining. WARN has cited the high rates of miscarriage and reproductive cancers among Lakota women as evidence of the adverse effects of uranium contamination (Unger 2004). The Pine Ridge reservation lies southeast of the Black Hills, which was the site of extensive uranium mining and milling during the 1940s–1970s. A series of studies traced gross alpha-radiation in groundwater and surface water sources in the Pine Ridge town of Red Shirt to the Edgemont uranium mill site (Jones 2011). Dozens of other abandoned mining and milling sites surround traditional Lakota territory, and the residents are currently fighting the expansion of uranium mining in the region in order to protect future generations.

## Akwesasne

The community that has perhaps been best studied is the Mohawk Nation at Akwesasne (located at the juncture of New York, Ontario, and Quebec), whose members traditionally derived most of their protein from fish from the St. Lawrence River and its tributaries. Three aluminum foundries were established upstream of Akwesasne, and all used PCBs as hydraulic fluids that leaked and contaminated the rivers, the fish, and consequently the people (Hwang et al. 1996). After analysis of fish by state and federal officials, tribal leaders advised the members to cease eating local fish in 1986. Although this has resulted in a decline in the levels of PCBs in breast milk and serum, PCB levels are still elevated compared with the general U.S. population (Fitzgerald et al. 1998). In addition, significantly higher levels of PCBs were found in Mohawk adolescents who were breastfed as infants (Gallo et al. 2011). Higher serum PCB concentrations were also associated with decrements in cognitive (Haase et al. 2009; Newman et al. 2009) and thyroid function (Schell et al. 2008) and elevated risk of diabetes (Codru et al. 2007), cardiovascular disease, and hypertension (Goncharov et al. 2008). Mohawk girls were more likely to have reached puberty at 12 years of age if they had higher serum PCBs (Denham et al. 2005), which could be due to the estrogenic effects of PCBs. Serum levels of PCBs in Mohawk men were associated with lower serum testosterone levels (Goncharov et al. 2009). Thus many aspects of Mohawk health may be adversely impacted by their exposure to PCBs.

## Environmental Justice, Indigenous People, and the Law

Indigenous communities are disproportionately exposed to environmental contaminants based on where they live and the cultural activities that put them in close contact with their environment. However, federal and state laws often make it easier for extractive and polluting enterprises to access tribal lands. Federal legislation and jurisprudence applicable to tribal lands are distinct from rules that apply to nontribal lands, and are typically inconsistent and inequitable. From Chief Justice John Marshall’s 1831 arbitrary definition of tribes as “domestic dependent nations” (Cherokee Nation *v*. State of Georgia 1831), to a 1985 Supreme Court decision that the Western Shoshone people lost title to their land because of “gradual encroachment” by the federal government (U.S. *v*. Dann 1985) (a concept that appears nowhere in the law before or since), federal courts and bureaucracies have long wielded language to constrain and derogate tribal peoples according to the political will of the day.

Because of these structural inequities, tribal jurisdictions are attractive to corporations seeking a lesser degree of environmental regulation, oversight, and enforcement than are imposed by state governments. Moreover, due to current social and structural inequalities, indigenous communities seeking environmental justice often experience barriers to their participation in prescribed environmental decision-making processes (Cole and Foster 2001).

Litigation under federal environmental laws and federal Indian law is fraught with challenges. Federal Indian law, a body of judge-made law arising mostly from litigation primarily before the United States Supreme Court, overwhelmingly denies environmental and cultural rights to Native American people. In addition, federal environmental legislation rarely recognizes environmental justice as a cause for action. Even when activists achieve victories in the courts, legislation and administrative agency rule makings can often undo years of environmental justice litigation. For these reasons, it is essential to develop policies that would better protect AI/AN communities from pollution, rather than leaving the matter to courts.

## Native Communities and Research

Because indigenous communities often do not have the legal, political, or economic means to resist the placement of polluting industries, indigenous people may suffer excess illness as a consequence of involuntary environmental exposures. However, because of historic antagonism to and distrust of non-native governments and academics, often these communities have not been studied to determine the extent of illness. In the past, some researchers entered indigenous communities with pre-developed projects, did not ask for community input, pressured residents into taking part in the studies, treated Natives as subjects and not colleagues, sensationalized problems in the community in their publications, used blood samples for unauthorized projects (Schnarch 2004), and did not give results to the community (Schell and Tarbell 1998).

These experiences have led some native communities to avoid engaging in research and others to make themselves available only to research projects that will include them as equal partners. The Akwesasne Mohawk developed an effective partnership with researchers, which resulted in > 50 published papers. Mohawk authors Arquette et al. (2002) highlighted the importance of continued collaborative research because of the need for better site- and Nation-specific data. This will provide tribal decision makers with specific information about contaminant levels in various local media and biota. These types of studies can also collect information about traditional cultural practices and natural resource use—information that can then be used to support the protection of natural resources and support the transfer of traditional knowledge and cultural practices to future generations.

To conduct such research, scientists and community members must develop equal and cooperative partnerships (Harding et al. 2012). Utilizing the community’s kinship network is important in garnering support for a study, recruiting study participants, and disseminating information. Especially important for the success of future environmental and reproductive health studies is increasing the number of indigenous midwives, physicians, and researchers who understand the potential health impacts of exposure to environmental contaminants.

## Environmental Reproductive Justice

Concerns about the community’s ability to reproduce, whether physically through the birth of healthy children or culturally through the passing on of traditional practices, has sparked interest in the need for environmental health research. As stated above, in Aamjiwnaang there was the noticeable decrease in male birth ratio (Mackenzie et al. 2005), which residents attribute to their proximity to petrochemical plants. At Akwesasne, a midwife pushed for health studies because of concerns of local mothers about the number of miscarriages in the community and the possibility of contaminated breast milk. Studies found that Mohawk women who ate local fish had higher levels of contaminants in their breast milk than a control group (Fitzgerald et al. 1998). Breastfeeding rates for AI/AN populations are well below the national average (Spieler 2010), an issue that health care providers are seeking to rectify. Indigenous mothers need to be confident that their breast milk is safe for their infants if these statistics are to be improved.

The reproductive capabilities of Mohawk women in Akwesasne are also affected by contamination; for example, PCB exposure has been associated with reducing the age of menarche in Mohawk girls (Denham et al. 2005). The Tewa Pueblo are concerned about the potential of birth defects connected to radiation exposure, and the women of Pine Ridge have attributed their high rates of miscarriage and reproductive organ cancers to contamination from uranium mines (Unger 2004). SLI Yupik people attribute their perceived high rate of cancer and other diseases to contamination from military sources and long-range transport (Carpenter et al. 2005; Henifin 2007; Miller and Riordan 1999).

In addition to concerns about the physical reproduction of community members, indigenous people are concerned about how environmental contamination impacts the reproduction of cultural knowledge. In Aamjiwnaang, oral traditions once passed down from grandfathers during fishing or grandmothers during berry picking and medicine gathering are being lost as those activities are no longer practiced because of concerns about these foods being contaminated. Rocks once used for sweat lodges are no longer being collected from local streams because the streams have become contaminated. The cedar used for making tea, smudging, and washing babies contains vanadium at concentrations as high as 6 mg/kg (ALS Laboratory Group Analytical Report, unpublished data), reflecting local releases to air of > 611 tons of vanadium between 2001 and 2010 (Environment Canada 2012). At Akwesasne, community members report a loss of language and culture around subsistence activities like fishing, which have been largely abandoned because of fears of exposure to contaminants. The generational reproduction of culturally informed interpersonal relationships has been affected as much as physical reproduction. We want to expand the definition of reproductive justice to include the capacity to raise children in culturally appropriate ways. For many indigenous communities, to reproduce culturally informed citizens requires a clean environment.

## Conclusion

Modern environmental law in North America is predicated on federal–state partnerships that did not initially account for pollution and environmental degradation of Native America (Grijalva 2011). Current regulatory gaps make it difficult to prevent and rectify environmental contamination that impacts AI/AN communities. This contamination threatens not only the health of indigenous communities, it also infringes on their reproductive rights, including the ability to impart cultural land-based knowledge to their children. Thus there is a great need for the concept of environmental reproductive justice in environmental health research. Continued research, involving collaborative partnerships among researchers, health care providers, and community members, is needed to determine the impact of environmental contamination on community members’ health and to develop necessary remediation, preventative measures, and protective policy interventions.
